# Estimating the number of undetected COVID-19 cases among travellers from mainland China

**DOI:** 10.12688/wellcomeopenres.15805.1

**Published:** 2020-06-15

**Authors:** Sangeeta Bhatia, Natsuko Imai, Gina Cuomo-Dannenburg, Marc Baguelin, Adhiratha Boonyasiri, Anne Cori, Zulma Cucunubá, Ilaria Dorigatti, Rich FitzJohn, Han Fu, Katy Gaythorpe, Azra Ghani, Arran Hamlet, Wes Hinsley, Daniel Laydon, Gemma Nedjati-Gilani, Lucy Okell, Steven Riley, Hayley Thompson, Sabine van Elsland, Erik Volz, Haowei Wang, Yuanrong Wang, Charles Whittaker, Xiaoyue Xi, Christl A. Donnelly, Neil M. Ferguson

**Affiliations:** 1 MRC Centre for Global Infectious Disease Analysis, Department of Infectious Disease Epidemiology, Imperial College London, London, UK; 2 Department of Statistics, University of Oxford, Oxford, UK

**Keywords:** Epidemiology, COVID-19, novel coronavirus, SARS-CoV-2, international

## Abstract

**Background:** Since the start of the COVID-19 epidemic in late 2019, there have been more than 152 affected regions and countries with over 110,000 confirmed cases outside mainland China.

**Methods: **We analysed COVID-19 cases among travellers from mainland China to different regions and countries, comparing the region- and country-specific rates of detected and confirmed cases per flight volume to estimate the relative sensitivity of surveillance in different regions and countries.

**Results: **Although travel restrictions from Wuhan City and other cities across China may have reduced the absolute number of travellers to and from China, we estimated that more than two thirds (70%, 95% CI: 54% - 80%, compared to Singapore; 75%, 95% CI: 66% - 82%, compared to multiple countries) of cases exported from mainland China have remained undetected.

**Conclusions: **These undetected cases potentially resulted in multiple chains of human-to-human transmission outside mainland China.

## Background

As of 18 ^th^ March 2020, over 80,000 cases of COVID-19 (formerly 2019-nCoV) have been reported in China (with 3231 deaths), and more than 110,000 cases have been detected in 152 regions and countries outside mainland China (including Hong Kong SAR and Macau SAR) ^1^. Several analyses have been undertaken to predict or estimate the risk of exported cases by country on the basis of flight connections between Wuhan City, China or mainland China as a whole and other regions and countries ^2– 6^. In this analysis we built on published work ^4^ to analyse COVID-19 cases reported and confirmed in different countries that were exported from mainland China, comparing the region- and country-specific rates of detected cases per flight volume to estimate the relative sensitivity of surveillance in different countries. We then estimate the number of COVID-19 cases exported from mainland China that have remained undetected worldwide.

## Methods

### Data sources

***Air traffic volume.*** Air travel data for the months of January, February, and March 2016 were obtained from the International Air Travel Association (IATA), with the sum divided by three to get destination-region- and destination-country-specific monthly averages. These numbers were not scaled up to reflect recent growth in air travel because any constant scaling of the monthly averages would simply be absorbed into the *λ* estimate and not affect other results. Flows of passengers within mainland China were excluded from this analysis.

***Number of cases detected outside mainland China.*** We collated data on 3276 cases in international travelers from media reports and provincial and national department of health press releases up until 27 February 2020 ^1,7^. We defined a local transmission as any transmission that occurred outside mainland China (Hong Kong SAR and Macau SAR are considered outside mainland China for this analysis). We only consider cases that were not transmitted locally. That is, we only considered cases detected outside mainland China that had a travel history to China and arrived outside mainland China by air, excluding repatriation flights ( Table 1). Based upon these inclusion criteria, a total of 173 cases were included in our analysis. The earliest date of travel for the cases included in the analysis is 1 January 2020, and the latest date of travel is 25 February 2020.

**Table 1.** Number of cases detected outside mainland China with travel history to China.

Country	Travel History to Hubei	No Travel History to Hubei	Unknown Travel History within China	Total (cases with a travel history to China)	Travelled by air (not repatriation flight)	Travelled on repatriation flight
Australia	15	0	0	15	15	0
Belgium	1	0	0	1	0	1
Cambodia	1	0	0	1	1	0
Canada	7	1	0	8	8	0
Finland	1	0	0	1	1	0
France	5	0	1	6	6	0
Germany	2	0	0	2	2	0
Hong Kong SAR	12	3	0	15	3	0
India	2	0	1	3	3	0
Italy	3	0	0	3	3	0
Japan	24	4	0	28	28	0
Macau SAR	5	3	0	8	1	0
Malaysia	9	4	0	13	9	0
Nepal	1	0	0	1	1	0
Philippines	3	0	0	3	3	0
Singapore	21	3	0	24	23	1
South Korea	12	2	0	14	12	1
Sri Lanka	1	0	0	1	1	0
Sweden	1	0	0	1	1	0
Taiwan	8	0	0	8	7	0
Thailand	14	3	5	22	19	0
United Arab Emirates	0	0	6	6	6	0
United Kingdom	0	1	2	3	3	0
United States of America	10	2	1	13	13	0
Vietnam	4	0	0	4	4	0
Total	162	26	16	204	173	3

### Analysis

We assume that the number of exported cases in a country *i* is Poisson distributed with a mean that depends on the air traffic from Wuhan to *i*, and the sensitivity of surveillance in *i* relative to a country *j*, denoted by *s _ei_*. For each country *i*, let *X _i_* be the number of exported cases (a count) and let *F _i_* be the volume of air traffic from Wuhan to country *i*. We can then write a joint log likelihood for the data from countries *i* and *j*:

$$l=X_{i}\;\text{ln}\left(s_{ei}\lambda F_{i}\right)-s_{ei}\lambda F_{i}+X_{j}\;\text{ln}\left(\lambda F_{j}\right)-\lambda F_{j}$$

ignoring additive constants. Thus, the maximum likelihood estimates for *λ* and *s _ei_* are:

$$\hat{\lambda }=\frac{X_{j}}{F_{j}}\;\text{and}\;{\hat{s}}_{ei}=\frac{X_{i}\;F_{j}}{X_{j}\;F_{i}}.$$

The likelihood-based confidence intervals are obtained by calculating the maximum log likelihood (over values of *λ*) for each value of *s _ei_*. Then the 95% confidence interval includes all those values of *s _ei_* such that $2\left(l_{\hat{s_{e\iota }}}-l_{s_{ei}}\right)\leq 3.84$ (the 95 ^th^ centile of the chi-squared distribution with 1 degree of freedom). These calculations were all performed using R version 3.6.0.

The relative sensitivities can also be estimated relative to *J* countries simultaneously using a method similar to above but with the log likelihood:

$$l=X_{i}\;\text{ln}\left(s_{ei}\lambda F_{i}\right)-s_{ei}\lambda F_{i}+\sum_{j=1}^{J}X_{j}\;\text{ln}\left(\lambda F_{j}\right)-\lambda F_{j}$$

Expected values can then be calculated for every country *i* as simply $\hat{\lambda }F_{i},$ and the expected value for all countries $\hat{\lambda }\Sigma_{j=1}^{N}F_{j},$ where N is the total number of countries with air traffic from Wuhan Tianhe International Airport (N = 119).

## Results

**Figure 1.**
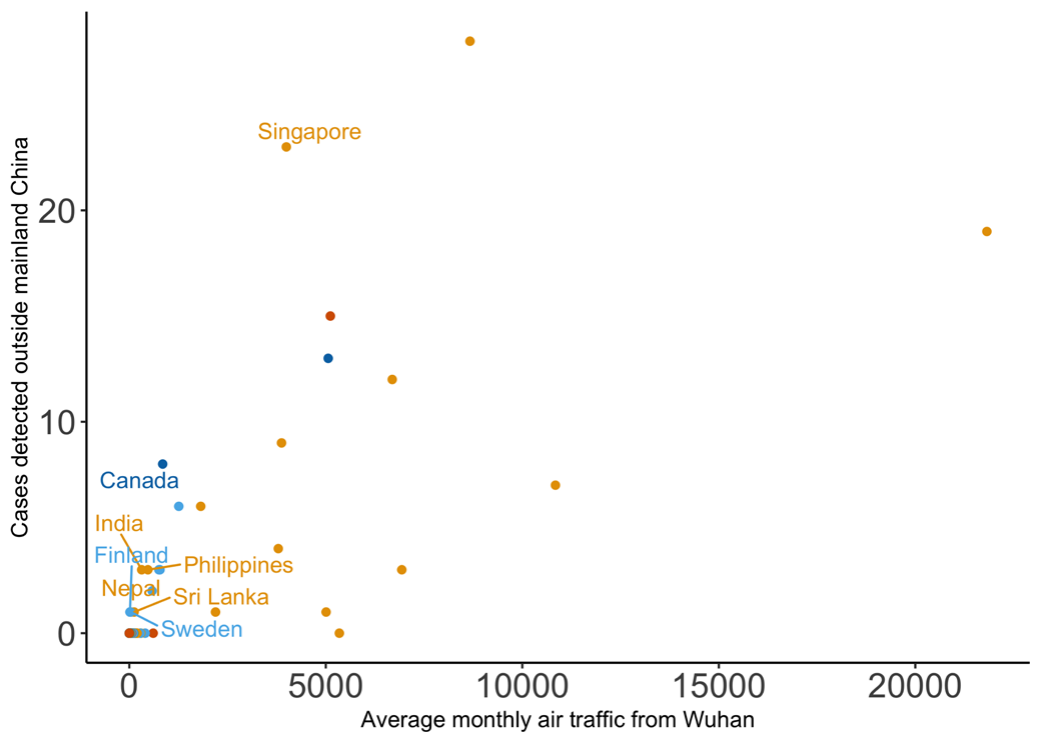
Exported COVID-19 cases vs average air traffic from Wuhan Tianhe International Airport by destination. The number of exported COVID-19 cases detected by region and country plotted against the average monthly international air traffic volume from Wuhan Tianhe International Airport aggregated by destination country. The colour of the points denotes the continent of the destination country (Asia - orange, Europe - light blue, Africa - green, North America - dark blue, South America - pink, and Oceania - dark orange).

The number of exported cases by country was plotted as a function of the average monthly passenger volume originating from Wuhan Tianhe International Airport on international flights ( Figure 1 ^7^). This showed Singapore to be an outlier in terms of having relatively many exported cases compared to the measure of air traffic volume.

The relative sensitivity of surveillance in individual countries was estimated compared to Singapore. Finland, Nepal, Philippines, Sweden, India, Sri Lanka, and Canada were all found to have relative sensitivity estimates greater than 1 (i.e. more cases were detected per passenger flight than in Singapore). Thus, a second set of relative sensitivity estimates was obtained for all other individual countries compared simultaneously to Singapore, Finland, Nepal, Philippines, Sweden, India, Sri Lanka, and Canada.

**Figure 2.**
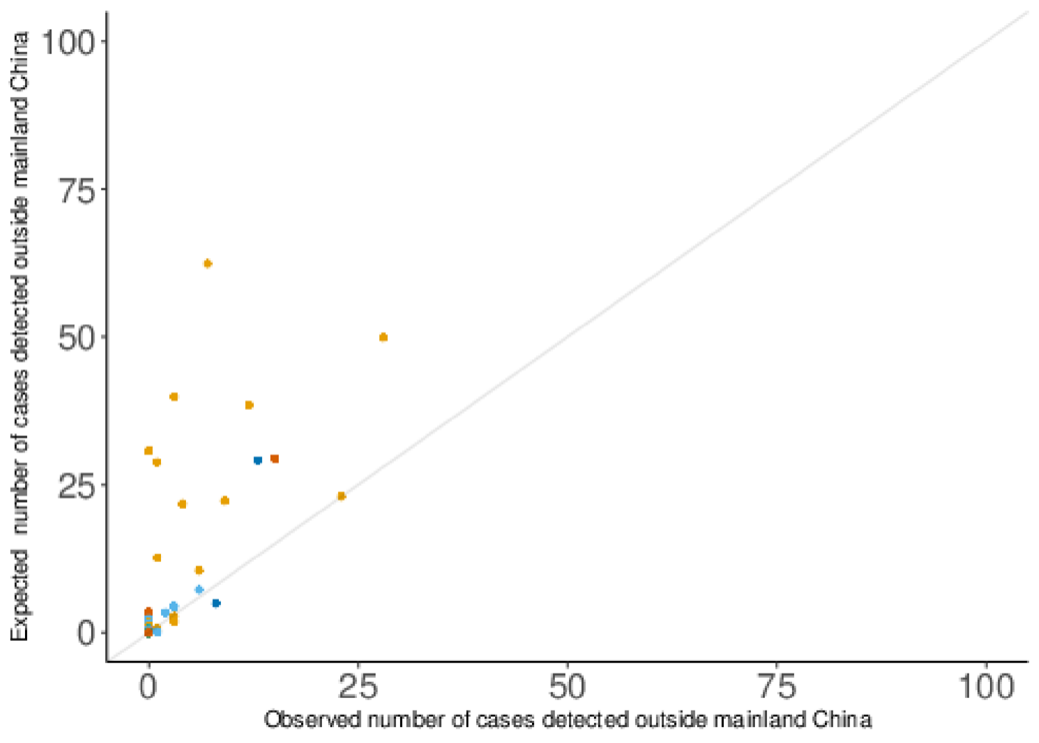
The expected and observed numbers of exported COVID-19 cases by country, relative to Singapore only. Values above the diagonal line indicate more cases were expected than were observed. The colour of the points denotes the continent of the destination country (Asia - orange, Europe - light blue, Africa - green, North America - dark blue, South America - pink, and Oceania - dark orange).

The region- and country-specific expected numbers of exported COVID-19 cases were in several cases substantially higher than the numbers detected ( Figure 2 ^7^). The sum of the expected numbers of exported COVID-19 cases for all regions and countries other than mainland China was 576.8 (95% CI: 372.2 - 845.4), based on the analysis relative to Singapore only, and 704.4 (95% CI: 510.3 - 942.3), based on the analysis relative to Singapore, Finland, Nepal, Philippines, Sweden, India, Sri Lanka, and Canada. Given that 173 such cases were detected, these central estimates suggest that between 70% (95% CI: 54% - 80%, relative to Singapore only) and 75% (95% CI: 66% - 82%, relative to Singapore, Finland, Nepal, Philippines, Sweden, India, Sri Lanka, and Canada) remained undetected.

## Discussion

Consistent with similar analyses ^4^, we estimated that more than two thirds of COVID-19 cases exported from mainland China have remained undetected worldwide, potentially leaving sources of human-to-human transmission unchecked (70%, 95% CI: 54% - 80% and 75%, 95% CI: 66% - 82%, undetected, based on comparisons to Singapore only and to Singapore, Finland, Nepal, Philippines, Sweden, India, Sri Lanka, and Canada, respectively). Undoubtedly, the exported cases vary in the severity of their clinical symptoms, making some cases more difficult to detect than others. However, some countries have detected significantly fewer than would have been expected based on the volume of flight passengers arriving from Wuhan City, China. These undetected cases potentially resulted in multiple chains of human-to-human transmission outside mainland China.

## Data availability

### Source data

The air travel data used in this analysis can be purchased from International Air Transport Association (IATA) via the following link: https://www.iata.org/en/services/statistics/air-transport-stats/.

### Underlying data

Zenodo: mrc-ide/COVID19_surveillance_sensitivity: Data and code used for submission. http://doi.org/10.5281/zenodo.3736643 ^7^.

This project contains the following underlying data:

- exported_cases.csv(information on the date of report, country of report and travel history of 3,276 cases outside mainland China)

### Extended data

Zenodo: mrc-ide/COVID19_surveillance_sensitivity: Data and code used for submission. http://doi.org/10.5281/zenodo.3736643 ^7^.

This project contains the following extended data:

- data_processing.R (R code to post-process international case data)

Data are available under the terms of the Creative Commons Zero "No rights reserved" data waiver (CC0 1.0 Public domain dedication).

## Footnotes

^1^This has been updated since the analysis presented here was released as a public report by the Imperial College London Coronavirus Response Team on available 22nd February 2020. This report is available at https://www.imperial.ac.uk/mrc-global-infectious-disease-analysis/covid-19/report-6-international-surveillance/. See https://doi.org/10.5281/zenodo.3736643.
