# Estimating the number of undetected COVID-19 cases among travellers from mainland China

**DOI:** 10.12688/wellcomeopenres.15805.2

**Published:** 2021-09-13

**Authors:** Sangeeta Bhatia, Natsuko Imai, Gina Cuomo-Dannenburg, Marc Baguelin, Adhiratha Boonyasiri, Anne Cori, Zulma Cucunubá, Ilaria Dorigatti, Rich FitzJohn, Han Fu, Katy Gaythorpe, Azra Ghani, Arran Hamlet, Wes Hinsley, Daniel Laydon, Gemma Nedjati-Gilani, Lucy Okell, Steven Riley, Hayley Thompson, Sabine van Elsland, Erik Volz, Haowei Wang, Yuanrong Wang, Charles Whittaker, Xiaoyue Xi, Christl A. Donnelly, Neil M. Ferguson

**Affiliations:** 1 MRC Centre for Global Infectious Disease Analysis, Department of Infectious Disease Epidemiology, Imperial College London, London, UK; 2 Department of Statistics, University of Oxford, Oxford, UK

**Keywords:** Epidemiology, COVID-19, novel coronavirus, SARS-CoV-2, international

## Abstract

**Background:** As of August 2021, every region of the world has been affected by the COVID-19 pandemic, with more than 196,000,000 cases worldwide.

**Methods: **We analysed COVID-19 cases among travellers from mainland China to different regions and countries, comparing the region- and country-specific rates of detected and confirmed cases per flight volume to estimate the relative sensitivity of surveillance in different regions and countries.

**Results: **Although travel restrictions from Wuhan City and other cities across China may have reduced the absolute number of travellers to and from China, we estimated that up to 70% (95% CI: 54% - 80%) of imported cases could remain undetected relative to the sensitivity of surveillance in Singapore. The percentage of undetected imported cases rises to 75% (95% CI 66% - 82%) when comparing to the surveillance sensitivity in multiple countries.

**Conclusions: **Our analysis shows that a large number of COVID-19 cases remain undetected across the world. ****These undetected cases potentially resulted in multiple chains of human-to-human transmission outside mainland China.

## Background

As of August 2021, over 196,000,000 cases of COVID-19 have been reported across the world with over 4,000,000 deaths ^1^. Several analyses have been undertaken to predict or estimate the risk of exported cases by country on the basis of flight connections between Wuhan City, China or mainland China as a whole and other regions and countries ^2– 8^. Salazar *et al.* ^4^, for instance, fit the number of reported cases in high surveillance countries and report that countries in Southeast Asia such as Indonesia and Thailand had reported fewer imported cases than expected despite a high volume of air travel with China. In this analysis we built on published work ^4^ to analyse COVID-19 cases reported and confirmed in different countries that were exported from mainland China, comparing the region- and country-specific rates of detected cases per flight volume to estimate the relative sensitivity of surveillance in different countries. We then estimate the number of COVID-19 cases exported from mainland China that have remained undetected worldwide.

## Methods

### Data sources

***Air traffic volume.*** Air travel data for the months of January, February, and March 2016 were obtained from the International Air Travel Association (IATA), with the sum divided by three to get destination-region- (Hong Kong SAR and Macau SAR) and destination-country-specific monthly averages. The data from 2016 were the most recent data to which we had access. These numbers were not scaled up to reflect recent growth in air travel because any constant scaling of the monthly averages would simply be absorbed into the estimates of model parameters (see Analysis) and not affect other results. Flows of passengers within mainland China were excluded from this analysis.

***Number of cases detected outside mainland China.*** We collated data on 3276 cases in international travelers from media reports and provincial and national department of health press releases up until 27 February 2020 ^1^ ^9^. Media reports on new cases of COVID-19 were followed daily from 15 ^th^ January 2020 to 27 ^th^ February 2020. Where possible, the details reported in the news were validated against official sources. Relevant websites such as ministries of health or local news media were identified through web searches. Reports in languages other than English were translated into English using translation services available online (e.g. Google translate). We defined a local transmission as any transmission that occurred outside mainland China (Hong Kong SAR and Macau SAR are considered outside mainland China for this analysis). We only consider cases that were not transmitted locally. That is, we only considered cases detected outside mainland China that had a travel history to China and arrived outside mainland China by air, excluding repatriation flights ( Table 1). Everyone we classified as a "case detected overseas" had the mode of travel either explicitly mentioned as air, or implied as the most probable mode of travel from mainland China to the destination (e.g. from China to Italy). Where multiple modes of travel are possible e.g. from mainland China to Hong Kong, we have only classified individuals as cases detected overseas where the mode of travel was explicitly mentioned as air. In most instances, all or most of the passengers on repatriation flights had been tested for the presence of SARS-CoV2. The cases detected through surveillance of repatriation flights are therefore not representative of the general sensitivity of surveillance in a country. We have therefore excluded these from the analysis.

**Table 1.** Number of cases detected outside mainland China with travel history to China.

Country	Travel History to Hubei	No Travel History to Hubei	Unknown Travel History within China	Total (cases with a travel history to China)	Travelled by air (not repatriation flight)	Travelled on repatriation flight
Australia	15	0	0	15	15	0
Belgium	1	0	0	1	0	1
Cambodia	1	0	0	1	1	0
Canada	7	1	0	8	8	0
Finland	1	0	0	1	1	0
France	5	0	1	6	6	0
Germany	2	0	0	2	2	0
Hong Kong SAR	12	3	0	15	3	0
India	2	0	1	3	3	0
Italy	3	0	0	3	3	0
Japan	24	4	0	28	28	0
Macau SAR	5	3	0	8	1	0
Malaysia	9	4	0	13	9	0
Nepal	1	0	0	1	1	0
Philippines	3	0	0	3	3	0
Singapore	21	3	0	24	23	1
South Korea	12	2	0	14	12	1
Sri Lanka	1	0	0	1	1	0
Sweden	1	0	0	1	1	0
Taiwan	8	0	0	8	7	0
Thailand	14	3	5	22	19	0
United Arab Emirates	0	0	6	6	6	0
United Kingdom	0	1	2	3	3	0
United States of America	10	2	1	13	13	0
Vietnam	4	0	0	4	4	0
Total	162	26	16	204	173	3

Based upon these inclusion criteria, a total of 173 cases were included in our analysis. The earliest date of travel for the cases included in the analysis is 1 January 2020, and the latest date of travel is 25 February 2020.

### Analysis

We assume that the observed number of exported cases in a country *i* is Poisson distributed with a mean that depends on the air traffic from Wuhan to *i*, and the sensitivity of surveillance in *i* relative to a country *j*, denoted by *s _ij_*. For each country *i*, let *X _i_* be the number of exported cases (a count) and let *F _i_* be the volume of air traffic from Wuhan to country *i*. We can then write a joint log likelihood for the data from countries *i* and *j*:

$$l=X_{i}\text{ln}\;(s_{ij}\lambda_{j}F_{i})\;\;-\;s_{ij}\lambda_{j}F_{i}\;+\;X_{j}\text{ln}\;(\lambda_{j}F_{j})\;-\lambda_{j}F_{j}$$

ignoring additive constants. Thus, the maximum likelihood estimates for *λ _j_* and *s _ij_* are:

$$\hat{\lambda_{j}}=\frac{X_{j}}{F_{j}}\;\;\text{and}\;s_{ij}=\frac{X_{i}F_{j}}{X_{j}F_{i}}$$

The likelihood-based confidence intervals are obtained by calculating the maximum log likelihood (over values of *λ _j_*) for each value of *s _ij_*. Then the 95% confidence interval includes all those values of *s _ij_* such that 2 ( $l_{\hat{s}ij}$ – *l _sij_*) ≤ 3.84 (the 95 ^th^ centile of the chi-squared distribution with 1 degree of freedom). These calculations were all performed using R version 3.6.0.

The relative sensitivities can also be estimated relative to *J* countries simultaneously using a method similar to above but with the log likelihood:

$$l=X_{i}\ln\;(s_{iJ}\lambda_{J}F_{i})-s_{iJ}\lambda_{J}F_{i}+\sum_{j=1}^{J}\left(X_{j}\ln\;(\lambda_{j}F_{j})\;-\;\lambda_{i}F_{j}\right)$$

Expected values can then be calculated for every country *i* as simply *λ _J_* F _i_, and the expected value for all countries is ${\hat{\lambda }}_{j}\;\sum_{i=1}^{N}\;F_{j}$ where N is the total number of countries with air traffic from Wuhan Tianhe International Airport (N = 119).

## Results

**Figure 1.**
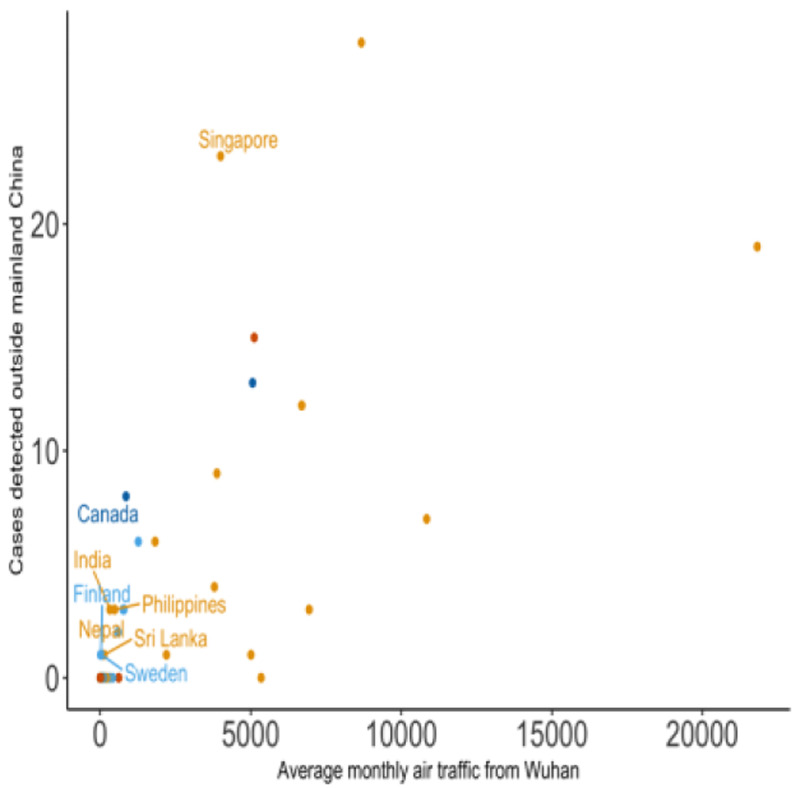
Exported COVID-19 cases vs average air traffic from Wuhan Tianhe International Airport by destination. The number of exported COVID-19 cases detected by region and country plotted against the average monthly international air traffic volume from Wuhan Tianhe International Airport aggregated by destination country. The colour of the points denotes the continent of the destination country (Asia - orange, Europe - light blue, Africa - green, North America - dark blue, South America - pink, and Oceania - dark orange).

The observed number of exported cases by country was plotted as a function of the average monthly passenger volume originating from Wuhan Tianhe International Airport on international flights ( Figure 1 ^9^). This showed Singapore to be an outlier in terms of having relatively many observed exported cases compared to the measure of air traffic volume.

The relative sensitivity of surveillance in individual countries was estimated compared to Singapore. Finland, Nepal, Philippines, Sweden, India, Sri Lanka, and Canada were all found to have relative sensitivity estimates greater than 1 (i.e. more cases were detected per passenger flight than in Singapore). Thus, a second set of relative sensitivity estimates was obtained for all other individual countries compared simultaneously to Singapore, Finland, Nepal, Philippines, Sweden, India, Sri Lanka, and Canada.

**Figure 2.**
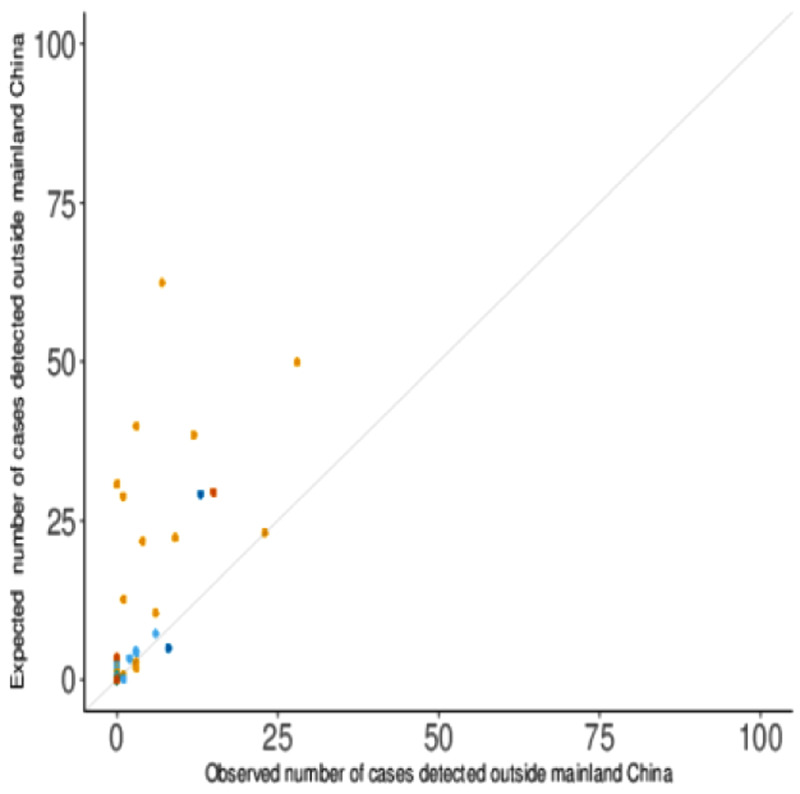
The expected and observed numbers of exported COVID-19 cases by country, with surveillance sensitivity relative to Singapore only.

The region- and country-specific expected numbers of exported COVID-19 cases were in several cases substantially higher than the numbers detected ( Figure 2 ^9^). The sum of the expected numbers of exported COVID-19 cases for all regions and countries other than mainland China was 576.8 (95% CI: 372.2 - 845.4), based on the analysis relative to Singapore only, and 704.4 (95% CI: 510.3 - 942.3), based on the analysis relative to Singapore, Finland, Nepal, Philippines, Sweden, India, Sri Lanka, and Canada. Given that 173 such cases were detected, these central estimates suggest that between 70% (95% CI: 54% - 80%, relative to Singapore only) and 75% (95% CI: 66% - 82%, relative to Singapore, Finland, Nepal, Philippines, Sweden, India, Sri Lanka, and Canada) remained undetected.

Values above the diagonal line indicate more cases were expected than were observed. The colour of the points denotes the continent of the destination country (Asia - orange, Europe - light blue, Africa - green, North America - dark blue, South America - pink, and Oceania - dark orange).

## Discussion

Consistent with similar analyses ^4, 10^, we estimated that more than two thirds of COVID-19 cases exported from Wuhan have remained undetected worldwide, potentially leaving sources of human-to-human transmission unchecked (70%, 95% CI: 54% - 80% and 75%, 95% CI: 66% - 82%, undetected, based on comparisons to Singapore only and to Singapore, Finland, Nepal, Philippines, Sweden, India, Sri Lanka, and Canada, respectively).

A limitation of our study is that we do not take into account the changes in air travel due to the travel advisories and restrictions imposed by various governments (though only those in force before 27 February 2020 would be relevant), which may have changed the volume of passengers flying into particular countries. Further, in using the data from 2016, we assume that the passenger volumes in early 2020 into each country is scaled by a constant factor. Access to more recent data on the changes in the number of passengers would likely improve the estimates of the sensitivity of surveillance presented here. For countries/regions that are connected to Wuhan using multiple modes of transport such as train links and water routes e.g., Hong Kong, surveillance is likely to have been enhanced at ports of entry other than airports. If so, the estimate of the sensitivity of surveillance as estimated here would therefore likely present an underestimate for these regions.

During the period of this study, Wuhan was the epicenter of the outbreak. Hence, it was reasonable to assume that a case detected outside China with travel history to Hubei province in this period is likely to be an imported case. However, epidemiological investigations are critical to ascertain the origin of a case. Timely public release of the results of such investigations could help public health professionals better assess the spread of the disease.

 Undoubtedly, the exported cases vary in the severity of their clinical symptoms, making some cases more difficult to detect than others. However, some countries have detected significantly fewer than would have been expected based on the volume of flight passengers arriving from Wuhan City, China. These undetected cases potentially resulted in multiple chains of human-to-human transmission outside mainland China.

## Data availability

### Source data

The air travel data used in this analysis can be purchased from International Air Transport Association (IATA) via the following link: https://www.iata.org/en/services/statistics/air-transport-stats/.

### Underlying data

Zenodo: mrc-ide/COVID19_surveillance_sensitivity: Data and code used for submission. http://doi.org/10.5281/zenodo.3736643 ^9^.

This project contains the following underlying data:

• exported_cases.csv(information on the date of report, country of report and travel history of 3,276 cases outside mainland China)

### Extended data

Zenodo: mrc-ide/COVID19_surveillance_sensitivity: Data and code used for submission. http://doi.org/10.5281/zenodo.3736643 ^9^.

This project contains the following extended data:

• data_processing.R (R code to post-process international case data)

Data are available under the terms of the Creative Commons Zero "No rights reserved" data waiver (CC0 1.0 Public domain dedication).

## Footnotes

^1^This has been updated since the analysis presented here was released as a public report by the Imperial College London Coronavirus Response Team on available 22nd February 2020. This report is available at https://www.imperial.ac.uk/mrc-global-infectious-disease-analysis/covid-19/report-6-international-surveillance/. See https://doi.org/10.5281/zenodo.3736643.
