# Supplementary material for: Estimating the number of undetected COVID-19 cases among travellers from mainland China
Source: Wellcome Open Res. 2021 Dec 6;5:143. Originally published 2020 Jun 15. [Version 3] doi: 10.12688/wellcomeopenres.15805.3 (PMC8477353; doi:10.12688/wellcomeopenres.15805.3)
Supplement: Peer review history

# Peer review: Reviewer response for version 2

**DOI:** 10.21956/wellcomeopenres.18948.r45821

**Published:** 2021-11-08

**Authors:** Juliet R.C. Pulliam, Jeremy Bingham

**Affiliations:** 1 South African DSI-NRF Centre of Excellence in Epidemiological Modelling and Analysis (SACEMA), Stellenbosch University, Stellenbosch, South Africa

The authors have addressed most of our concerns, except that the article still links to the old version of the code. We note that there does appear to be updated code available via the GitHub repository* that is linked from the Zenodo page, but some of the changes referred to in the response (eg updates to the README) do not seem to be available. We recommend that the authors re-check that all updates have been pushed to the repository and request that a direct link to the updated code be provided.

 * https://github.com/mrc-ide/COVID19_surveillance_sensitivity

Is the work clearly and accurately presented and does it cite the current literature?

Yes

If applicable, is the statistical analysis and its interpretation appropriate?

Yes

Are all the source data underlying the results available to ensure full reproducibility?

Partly

Is the study design appropriate and is the work technically sound?

Yes

Are the conclusions drawn adequately supported by the results?

Yes

Are sufficient details of methods and analysis provided to allow replication by others?

Partly

Reviewer Expertise:

infectious disease epidemiology and modelling

We confirm that we have read this submission and believe that we have an appropriate level of expertise to confirm that it is of an acceptable scientific standard.

# Response

**Published:** 2021-11-19

**Authors:** Christl Donnelly

**Affiliations:** Imperial College London, UK

We thank the reviewer for highlighting that the Zenodo link pointed to an older version of the code. We have now added a link to the latest version of the repository. We have also added additional instructions to the README file to help the readers replicate the analysis.

# Peer review: Reviewer response for version 2

**DOI:** 10.21956/wellcomeopenres.18948.r45820

**Published:** 2021-10-06

**Authors:** Sebastian Funk

**Affiliations:** 1 Centre for Mathematical Modelling of Infectious Diseases, London School of Hygiene & Tropical Medicine, London, UK

Thanks for the revisions, which I think have improved the article.

 As a last point, can I just ask to clarify that you can't add a column with the source (website?) for the data table. Was there no records kept of this? I couldn't find the answer to this question in the response.

Is the work clearly and accurately presented and does it cite the current literature?

Partly

If applicable, is the statistical analysis and its interpretation appropriate?

Yes

Are all the source data underlying the results available to ensure full reproducibility?

Partly

Is the study design appropriate and is the work technically sound?

Yes

Are the conclusions drawn adequately supported by the results?

Yes

Are sufficient details of methods and analysis provided to allow replication by others?

Partly

Reviewer Expertise:

infectious disease dynamics

I confirm that I have read this submission and believe that I have an appropriate level of expertise to confirm that it is of an acceptable scientific standard.

# Response

**Published:** 2021-10-14

**Authors:** Christl Donnelly

**Affiliations:** Imperial College London, UK

Thanks for your review of our manuscript. 

 We have now uploaded to GitHub a list of the many sources consulted to identify COVID-19 cases among travellers. 

 list_of_sources_consulted.csv

COVID19_surveillance_sensitivity/list_of_sources_consulted.csv at bf210752a493a61bc230cfd21d10337c6de70fb4 · mrc-ide/COVID19_surveillance_sensitivity · GitHub

# Peer review: Reviewer response for version 2

**DOI:** 10.21956/wellcomeopenres.18948.r45822

**Published:** 2021-09-27

**Authors:** Hannah E. Clapham

**Affiliations:** 1 Saw Swee Hock School of Public Health, National University of Singapore and National University Health System, Singapore, Singapore

No further comments.

Is the work clearly and accurately presented and does it cite the current literature?

Partly

If applicable, is the statistical analysis and its interpretation appropriate?

Yes

Are all the source data underlying the results available to ensure full reproducibility?

Yes

Is the study design appropriate and is the work technically sound?

Yes

Are the conclusions drawn adequately supported by the results?

Yes

Are sufficient details of methods and analysis provided to allow replication by others?

Partly

Reviewer Expertise:

Infectious disease epidemiology and dynamics

I confirm that I have read this submission and believe that I have an appropriate level of expertise to confirm that it is of an acceptable scientific standard.

# Peer review: Reviewer response for version 1

**DOI:** 10.21956/wellcomeopenres.17332.r39749

**Published:** 2020-08-13

**Authors:** Hannah E. Clapham

**Affiliations:** 1 Saw Swee Hock School of Public Health, National University of Singapore and National University Health System, Singapore, Singapore

This is a well-done analysis that estimates the number of unreported cases that were imported from China early on in the pandemic. It does this using data on air travel volume between China and other countries, and the reported numbers of cases in the places that reported the highest numbers of cases given their air traffic volume. This analysis was highly relevant early in the pandemic as infections spread from China.

 I have a few comments on points for clarification below.

 Abstract:

 The statement of the results about 70/75%... in the abstract is confusing. Suggest rephrasing.

 Conclusion: I wonder if this is a conclusion from the paper. I would suggest the addition here that the analysis leads to estimates that there were many unreported imported infections, and that potentially lead to transmission.

 Main text:

 Background: It would be helpful to have a statement about what the previous analysis in reference 4 did/showed.

 Methods: Please add more detail on how airports in China were used in the flow calculation, and also on the definition of a destination region. In the results section initially Wuhan is focused on but the general conclusions seem to be from all of mainland China. Please clarify throughout.

 Please add more detail from where the collated data on imported cases was obtained.

 Were all excluded cases excluded because they were defined as local or due to missing information on this?

 Was data available on which location was travelled from within China for the imported cases? If not, how was this dealt with in the analysis?

 Results:

 Figure 2 legend, are the numbers shown relative to Singapore numbers, or is the analysis done relative to Singapore and then the estimates of imported cases shown? At the moment, the legend reads as the former, but my understanding of the analysis is that is it the latter. Please clarify.

 Discussion: Please add on limitations of the analysis, in particular how this relates to the available data including classification as imported vs local, and that this data needed to be publicly available.

Is the work clearly and accurately presented and does it cite the current literature?

Partly

If applicable, is the statistical analysis and its interpretation appropriate?

Yes

Are all the source data underlying the results available to ensure full reproducibility?

Yes

Is the study design appropriate and is the work technically sound?

Yes

Are the conclusions drawn adequately supported by the results?

Yes

Are sufficient details of methods and analysis provided to allow replication by others?

Partly

Reviewer Expertise:

Infectious disease epidemiology and dynamics

I confirm that I have read this submission and believe that I have an appropriate level of expertise to confirm that it is of an acceptable scientific standard, however I have significant reservations, as outlined above.

# Response

**Published:** 2021-08-12

**Authors:** Christl Donnelly

**Affiliations:** Imperial College London, UK

1. The statement in the abstract has been rephrased for clarity.
2. The conclusion in the abstract has been edited as suggested.
3. We have added a sentence about the analysis and conclusions of Reference 4.
4. Destination-regions refer to Hong Kong SAR and Macau SAR, this clarification has been added to the text.
5. We have now edited the Results and the Discussion sections to emphasise that we are estimating the number of exported cases from Wuhan (rather than China).
6. We have expanded the section on data collation to clarify the points raised by the reviewer. The inclusion criteria have been elaborated to clarify when a case was excluded.
7. As the reviewer has rightly noted, the numbers in figure 2 are the estimates of imported cases. The legend has been edited to reflect this.
8. We have expanded the discussion to highlight the limitations and potential biases of the analysis.

# Peer review: Reviewer response for version 1

**DOI:** 10.21956/wellcomeopenres.17332.r39133

**Published:** 2020-07-31

**Authors:** Sebastian Funk

**Affiliations:** 1 Centre for Mathematical Modelling of Infectious Diseases, London School of Hygiene & Tropical Medicine, London, UK

This manuscript aims to quantify the amount of underdetection of cases outside of China during the early phase of the COVID-19 pandemic. It assumes that the numbers of cases are centred around the product of the number of flights from Wuhan, an estimated per-country parameter \lambda, and an estimated relative per-country surveillance capacity.

 Overall the methodology is sensible and the findings of interest at the time.

 Comments:

- The manuscript is missing detail on how the 3276 cases were collated. Was this done systematically and if so how? Were non-English reports collated and was there any attempt to correct for language biases? Have you got references for the cases and, if yes, could they be added, e.g., to the csv file?
- The index could be more consistent, e.g. it seems \lambda=X_j/F_j should have an index j, and s should have indices i and j (and no e).
- It would be interesting to see the results here compared to Golding *et al.*, https://www.medrxiv.org/content/10.1101/2020.07.07.20148460v1, which had the same aim but used a different methodology/data - also, to compare to other estimates of underdetection in the relevant countries. ^1^
- A thorough discussion of potential biases/limitations is missing.

Is the work clearly and accurately presented and does it cite the current literature?

Partly

If applicable, is the statistical analysis and its interpretation appropriate?

Yes

Are all the source data underlying the results available to ensure full reproducibility?

Partly

Is the study design appropriate and is the work technically sound?

Yes

Are the conclusions drawn adequately supported by the results?

Yes

Are sufficient details of methods and analysis provided to allow replication by others?

Partly

Reviewer Expertise:

infectious disease dynamics

I confirm that I have read this submission and believe that I have an appropriate level of expertise to confirm that it is of an acceptable scientific standard, however I have significant reservations, as outlined above.

## References

[1] : Reconstructing the global dynamics of under-ascertained COVID-19 cases and infections. *medRxiv* .2020; 10.1101/2020.07.07.20148460 10.1101/2020.07.07.20148460 PMC757779633087179

# Response

**Published:** 2021-08-12

**Authors:** Christl Donnelly

**Affiliations:** Imperial College London, UK

1. The section on data collation has been expanded to clarify the points raised by the reviewer.
2. Thanks, we have edited the indices.
3. A direct comparison with the results of Golding et al is difficult because they present country-specific ascertainment estimates while the goal of our analysis was to estimate the number of undetected COVID-19 cases globally and we have therefore not provided per-country estimates of surveillance sensitivity.
4. We have expanded the discussion to highlight the limitations and potential biases of the analysis.

# Peer review: Reviewer response for version 1

**DOI:** 10.21956/wellcomeopenres.17332.r39131

**Published:** 2020-07-08

**Authors:** Juliet R.C. Pulliam, Jeremy Bingham

**Affiliations:** 1 South African DSI-NRF Centre of Excellence in Epidemiological Modelling and Analysis (SACEMA), Stellenbosch University, Stellenbosch, South Africa

Summary: Bhatia et al. estimate the numbers of undetected COVID-19 cases exported from Mainland China based on historical air traffic patterns and public data on COVID-19 cases imported to other countries that originated in China. The observed number of cases imported to each country is assumed to be Poisson distributed, and the expected number of imported cases relative to Singapore (used as a reference country because of its high ratio of detected cases per flight volume) is derived using standard maximum likelihood estimation methods. A similar procedure is used to compare the expected number of imported cases relative to a set of reference countries which had estimated sensitivities of surveillance higher than that of Singapore. The main finding is that at least 2/3 of cases exported from China remained undetected worldwide.

 Issues and comments:

Major

 The code provided does not run out-of-the-box. In data_processing.R `exported_cases_paper_test.csv` should be `exported_cases.csv` and the line defining the total number of exported cases should be moved after the definition of `exported`.

 Furthermore, only the data processing script for cases in international travelers is provided, not the code for data analysis or visualization of results.

 While we understand that the authors cannot share the IATA data, they should *at least* provide results sufficient to reproduce Figure 2, and preferably all code for analysis and visualization such that anyone with access to the data could reproduce the results.

Minor

 There is a disconnect between the use of passenger flow data from only WTIA and analysis of cases thought to have acquired infection in mainland China, regardless of whether they travelled through Wuhan or Hubei; some explanation of the decision to use these inconsistent definitions is warranted.

 Please clarify why repatriation flights have been excluded when selecting cases for analysis.

 At the beginning of the analysis section, “We assume that the number of exported cases in a country *i*…” should read “We assume that the observed number of exported cases in a country *i*…”. This distinction should be clarified throughout.

 Some mention could be made of the reason for using 2016 flight data rather than more recent data.

 Some mention of potential biases and pitfalls in methods and the data would be appropriate.

 There are a small number of relevant, recently published works not mentioned - e.g. https://www.medrxiv.org/content/10.1101/2020.03.23.20038331v2 ^1^  and https://doi.org/10.1016/S0140-6736(20)30411-6 ^2^.

 The background in the abstract is very out of date; suggest updating numbers and adding a date marker (‘as of…’ or similar)

 Additional labels for the figures would be useful (beyond the select countries labeled in Figure 1). Alternatively, per-country estimates could be made available as a table.

 In the methods section (‘Data Sources’) the parameter lambda is referred to before being introduced.

 In the discussion “Consistent with similar *analyses*” has only one citation.

Is the work clearly and accurately presented and does it cite the current literature?

Yes

If applicable, is the statistical analysis and its interpretation appropriate?

Yes

Are all the source data underlying the results available to ensure full reproducibility?

Partly

Is the study design appropriate and is the work technically sound?

Yes

Are the conclusions drawn adequately supported by the results?

Yes

Are sufficient details of methods and analysis provided to allow replication by others?

Partly

Reviewer Expertise:

infectious disease epidemiology and modelling

We confirm that we have read this submission and believe that we have an appropriate level of expertise to confirm that it is of an acceptable scientific standard, however we have significant reservations, as outlined above.

## References

[1] : Estimating the number of undetected COVID-19 cases exported internationally from all of China. *medRxiv* .2020; 10.1101/2020.03.23.20038331 10.1101/2020.03.23.20038331

[2] : Preparedness and vulnerability of African countries against importations of COVID-19: a modelling study. *The Lancet* .2020;395(10227) : 10.1016/S0140-6736(20)30411-6 871-877 10.1016/S0140-6736(20)30411-6 PMC715927732087820

# Response

**Published:** 2021-08-12

**Authors:** Christl Donnelly

**Affiliations:** Imperial College London, UK

1. We apologise for the omission of relevant code. We have now added all relevant code so that someone with relevant data can reproduce the results. We have also included a file with dummy data on travel volume to help the readers. The README file has also been updated to include instructions on running the code.
2. Everyone we classified as cases detected overseas had travel by air either explicitly mentioned, or implied as the most probable mode of travel from mainland China to the destination (e.g. from China to Italy). Where multiple modes of travel are possible e.g. from mainland China to Hong Kong, we have only classified individuals as cases detected overseas where the mode of travel was explicitly mentioned as air. The text has been updated to clarify this.
3. In most instances, all passengers on repatriation flights had been tested for the presence of SARS-CoV2. The cases detected through surveillance of repatriation flights are therefore not representative of the typical sensitivity of surveillance in a country. We have therefore excluded these from the analysis. The text has been updated to clarify this.
4. This sentence has now been edited and the distinction has been emphasised in the rest of the text.
5. The data from 2016 were the most recent data to which we had access when undertaking our analysis. Further, any constant scaling of the volume of passengers would not affect the estimates of model parameters (lambda and s_e). This has been emphasised in the text.
6. We have included the limitations of the method and data sources in the discussion.
7. Thanks for highlighting these relevant references. We have now included reference to these in the section Background.
8. The abstract and the reference were updated as of August 2021.
9. The reference to lambda has been removed from the methods section and a reference added to the appropriate section.
10. We have added reference to other studies conducted at the time which provide estimate surveillance sensitivity globally.
